# Circulating levels of gut hormones in anorexia nervosa before and after short-term weight restoration

**DOI:** 10.1016/j.pnpbp.2025.111576

**Published:** 2025-12-04

**Authors:** Theresa Kolb, Louisa Licht, Friederike I. Tam, Evelina M. Stender, Michaela Ohme, Alessandra Borsini, Stefan Ehrlich, Nikolaos Perakakis

**Affiliations:** a Division of Psychological and Social Medicine and Developmental Neurosciences, Faculty of Medicine, Technische Universität Dresden, Dresden, Germany; b Department of Internal Medicine III, University Hospital Carl Gustav Carus, Technische Universität Dresden, Fetscherstraße 74, 01307 Dresden, Germany; c Eating Disorder Treatment and Research Center, Department of Child and Adolescent Psychiatry, Faculty of Medicine, Technische Universität Dresden, Dresden, Germany; d Stress, Psychiatry and Immunology Laboratory, Institute of Psychiatry, Psychology and Neuroscience, Department of Psychological Medicine, King’s College London, London, UK; e Paul Langerhans Institute Dresden (PLID), Helmholtz Center Munich, University Hospital and Faculty of Medicine, TU Dresden, Fetscherstraße 74, 01307 Dresden, Germany; f German Center for Diabetes Research (DZD e.V.), Ingolstädter Landstrasse 1, 85764 Neuherberg, Germany

## Abstract

Gastrointestinal hormones such as glucagon-like peptide-1 (GLP-1), gastric inhibitory peptide (GIP), glucagon, and glicentin are important regulators of appetite and glucose homeostasis. While agonists of GLP-1 and GIP receptors are approved treatments for type 2 diabetes and obesity, their role in anorexia nervosa (AN) remains largely unknown.

In this study, we measured fasting serum levels of GLP-1, GIP, glucagon, and glicentin in 80 female patients with AN before (acAN-T1) and after short-term weight restoration (acAN-T2) compared to 80 age-matched female healthy controls (HC).

GIP levels were higher (42.9%) in acAN-T1 than in HC, while GLP-1, glicentin, and glucagon showed no group differences. Additionally, acAN-T1 patients exhibited lower fasting glucose (*−*8.4%) and insulin (*−*42.6%) levels than HC. In acAN-T2, GIP, GLP-1, and glicentin levels decreased (*−*30.4%, *−*9.7%, − 15.7 %, respectively), with only GIP normalizing. Glucose and insulin levels increased (4.5% and 41.4%, respectively), although they remained lower than in HC.

Importantly, changes in GIP levels after short-term weight restoration negatively correlated (*r* = *−*0.279) with changes in glucose levels. Furthermore, GIP levels in acAN-T1 were positively associated with disordered eating and depressive symptoms, independent of BMI-SDS.

These results reveal that GIP shows a distinct pattern of dysregulation and normalization in AN and link GIP levels to both glucose metabolism and symptom severity in AN. Thus, our findings support the rationale for investigating GIP receptor-targeted therapies in AN.

## 1. Introduction

Anorexia nervosa (AN) is a complex and life-threatening psychiatric disorder characterized by weight loss, commonly achieved through severe food restriction which is fuelled by body image distortion. The role of appetite-regulating hormones in the pathophysiology of AN, as well as the impact of this disorder on glucose metabolism, remain largely unknown.

Hormones such as glucagon-like peptide-1 (GLP-1), glucose-dependent insulinotropic polypeptide (GIP), glicentin, and glucagon play key roles in regulating appetite and glucose homeostasis (Drucker and Holst, 2023). Except for GIP, these hormones are all derived from the post translational processing of the proglucagon peptide. GLP-1 and GIP, both incretin hormones, are secreted in response to food intake and stimulate insulin secretion. They differentially modulate glucagon release through suppression (GLP-1) or stimulation (GIP) to regulate glucose homeostasis (Drucker and Holst, 2023; Holst, 2007). Glicentin is co-secreted with GLP-1 by the L-cells of the gut (Holst, 2007; Müller et al., 2019). Its specific physiological role is not fully understood. However, glicentin has been shown to stimulate insulin secretion and potentially to play a role in appetite and weight regulation as well as in glucose homeostasis, thus serving as another possible target for the treatment of metabolic diseases (Hoffmann et al, 2023; Holst, 2022; Perakakis et al., 2019, 2021; Perakakis et al., 2022b; Perakakis et al., 2022a; Perakakis and Mantzoros, 2020; Wewer Albrechtsen et al., 2023). Lastly, glucagon, primarily secreted by the pancreatic islets, is essential for maintaining glucose homeostasis by increasing blood glucose levels (Wewer Albrechtsen et al., 2023).

These hormones have been extensively studied in patients with energy excess. In obesity and type 2 diabetes mellitus (T2DM), monoagonists targeting the GLP-1 receptor (e.g., semaglutide, liraglutide) and a dual agonist of GLP-1 and GIP receptor (i.e. Tirzepatide) have been approved as treatment due to their profound effects on reducing body weight and glucose levels (Kusminski et al., 2024). Similarly, GLP-1/Glucagon as well as GLP-1/GIP/Glucagon receptor co-agonists are under evaluation in Phase III clinical trials for their potential use in the treatment of obesity and T2DM (Kusminski et al., 2024).

In contrast, research on the role of these hormones in states of severe energy deficiency, such as AN, is limited and has yielded contradictory findings. Specifically, early studies assessing GIP and GLP-1 levels in individuals with AN have reported conflicting results. Several studies showed lower GLP-1 fasting blood levels in individuals with AN in comparison to healthy control participants (Germain et al., 2007; Heruc et al., 2018; P. Tomasik et al., 2002; P. J. Tomasik et al., 2004), whereas other findings suggest no differences (Germain et al., 2007; Heruc et al., 2018). Regarding GIP, some studies found normal blood fasting levels (Alderdice et al., 1985; Dixon et al., 1985; Stock et al., 2005; P. J. Tomasik et al., 2004), while others reported elevated (P. J. Tomasik et al., 2004) or reduced levels (Stock et al., 2005). To our knowledge, no studies have investigated glicentin in AN, whereas only one previous study has measured glucagon in this disorder, reporting lower levels compared to healthy control participants (Kumai et al., 1988). An important limitation of the earlier studies was the use of outdated assays, which have been heavily criticised for their low accuracy due to high cross-reactivity between homologous peptides (Bak et al., 2014; Deacon and Holst, 2009; Wewer Albrechtsen et al., 2016). Further, the contradictory findings may be partially attributed to the relatively small sample sizes of the original studies.

Given the inconclusive previous evidence, this study aimed to compare the serum levels of our hormones of interest—GLP-1, GIP, glucagon, and glicentin—between patients with AN and HC. Our second aim was to evaluate whether short-term weight restoration in AN affects the levels of these hormones. Finally, our third aim was to investigate whether the changes in our hormones of interest observed in AN or during weight restoration treatment are associated with parameters of glucose homeostasis or clinical measures of psychopathology.

## 2. Experimental procedures

### 2.1. Participants

For 160 female participants, serum samples were available to be analysed in this study: 80 female underweight patients with acute AN (acAN-T1, 12.1–24.4 years old) and 80 HC (12.2–27.7 years old) age-matched to the acAN-T1 group. Patients with AN were admitted to intensive treatment of an eating disorder (ED) program at a child and adolescent psychiatry and psychosomatic medicine department of a tertiary care university hospital, and assessed within 96 h of admission to treatment (timepoint 1, acAN-T1). Diagnosis of AN was established using a modified version of the expert form of the Structured Interview for Anorexia and Bulimia Nervosa (SIAB-EX) (Fichter and Quadflieg, 2001) requiring a body mass index (BMI) below the 10th age percentile (if *<* 15.5 years) or below 17.5 kg/m^2^ (if *>* 15.5 years). 79 patients were reassessed after short-term weight restoration (timepoint 2, acAN-T2; BMI increase of at least 12% was an inclusion criterion) through higher calorie refeeding. HC had to be of normal weight, eumenorrhoeic, and without any history of mental illness and were recruited through advertisement among middle/high school and university students.

Information regarding exclusion criteria was obtained from all participants using the SIAB-EX (Fichter and Quadflieg, 2001), supplemented by our own semi-structured interview and medical records. Comorbid diagnoses were taken from medical records and confirmed by an expert clinician with over 10 years of experience. Participants of all groups were excluded if they had a history of any of the following diagnoses: organic brain syndrome, schizophrenia, substance dependence, psychosis not otherwise specified, bipolar disorder, bulimia nervosa, or binge-eating disorder. Further exclusion criteria for all participants were an intelligence quotient (IQ) below 85; current substance abuse; inflammatory, neurologic, or metabolic illness; chronic medical or neurological illness that could affect appetite, eating behaviour or body weight; clinically relevant anemia; pregnancy or breast feeding. Psychotropic medication within 4 weeks before the study was an additional exclusion criterion for all groups, with the exception of selective serotonin reuptake inhibitors in the acAN groups. All protocols received ethical approval by the local Institutional Review Board, and all participants (or if underage, their guardians) gave written informed consent. The study was performed in accordance with the ethical standards as laid down in the 1964 Declaration of Helsinki and its later amendments.

Study data were collected and managed using secure, web-based electronic data capture tools REDCap (Research Electronic Data Capture) (Harris et al., 2009, 2019).

### 2.2. Clinical measures

In addition to the evaluation with the SIAB-EX (Fichter and Quadflieg, 2001), eating disorder-specific psychopathology was assessed with the German version of the self-report questionnaire Eating Disorder Inventory-2 (EDI-2) (Paul and Thiel, 2005). Depressive symptoms were explored using the German version of the Beck Depression Inventory-II (BDI-II), while general levels of psychopathology and anxiety symptoms were evaluated using the revised Symptom Checklist 90 (SCL-90-R) (Franke, 2002). The IQ was estimated with short versions of the German adaptation of the Wechsler Adult Intelligence Scale (Wechsler Intelligenztest für Erwachsene) (Wechsler, 2006) or for study participants aged 15 years or younger the Wechsler Intelligence Scale for Children (Hamburg-Wechsler Intelligenztest für Kinder IV) (Petermann and Petermann, 2010). Additionally, to the BMI, the age-corrected BMI standard deviation score (BMI-SDS) was calculated (Hemmelmann et al., 2010; Kromeyer-Hauschild et al., 2001).

### 2.3. Procedure

Venous blood samples were collected between 7 and 9 a.m. after an overnight fast (for the acAN-T1 group within 96 h of admission to treatment). To yield blood serum for assessment of insulin, GIP, GLP-1, glicentin, and glucagon, samples were left to clot for 30 min at 6–8°C and then centrifuged (2500 ×*g*, 15 min, 5°C). To yield plasma for leptin assessment, aprotinin was added during blood sampling (270 KIU/ml final concentration) to prevent protein degradation by serine proteases and samples were immediately centrifuged (2500 ×*g*, 15 min, 5°C). All samples were aliquoted immediately after centrifugation and stored at −80°C. We used commercially available enzyme-linked immunoabsorbent assays to measure serum insulin, GIP, GLP-1, glicentin, and glucagon concentrations (ELISAs, Northern Lights Mercodia, Uppsala, Sweden) as well as leptin plasma concentrations (Leptin Human ELISA, Clinical Range, Biovendor, Brno, Czech Republic). Serum glucose levels were measured with a super GL compact device (Dr. Müller Gerätebau GmbH).

### 2.4. Statistical analysis

Statistical analyses were performed using SPSS Statistics (Version 27, *IBM SPSS Statistics for Windows*, 2023) and JASP (JASP Team, J, 2024, Version 0.18.3). Blood parameter concentrations, with the exception of glucose, exhibited a skewed distribution across all three groups. To address this, a logarithmic transformation (log10) was applied to insulin, GLP-1, GIP, glucagon, and glicentin values. Outliers defined as values larger than ± 3 standard deviations (SD) were adjusted by replacing them with the value of 3 SD of the respective parameter (Ruppert, 2014).

Homeostasis Model Assessment (HOMA) was employed to calculate insulin resistance (HOMA-IR) using the following equations:

$$\text{HOMA}\;-\;\text{IR}\;(\text{mmol}/\text{l})=\;\text{Fasting Insulin}\;{\left(\text{mU}/\text{l}\right)}^{*}\;\text{Fasting Glucose}\;(\text{mmol}/\text{l})/22.5$$

Subsequent to log10-transformation, we employed parametric tests for analysis. Cross-sectional differences in blood parameter concentrations between HC and acAN-T1 as well as between HC and acAN-T2 were examined using independent Student’s *t*-tests. We assessed longitudinal changes within the acAN group (acAN-T1, acAN-T2) with paired Student’s t-tests. Additionally, as part of a supplementary analysis, we conducted non-parametric tests to compare the original blood parameter data between groups: Mann-Whitney *U* test for group comparisons of acAN-T1 with HC and acAN-T2 with HC, and Wilcoxon signed-rank test for comparisons of acAN-T1 with acAN-T2. Bayesian independent sample t-tests were applied in a post-hoc fashion to investigate the amount of evidence for the normalization hypothesis, i.e. the absence of group differences between acAN-T2 and HC if initially altered hormone concentrations showed significant changes with weight restoration based on frequentist statistics. Spearman correlation coefficients were calculated for blood parameters of interest that showed significant group differences (alterations in acAN-T1 compared to HC, changes between acAN-T1 and acAN-T2). Statistical significance was determined as *P <* 0.05. *P* values were adjusted for multiple comparisons using False Discovery Rate (FDR) correction method of Benjamin and Hochberg (1995) where appropriate.

In exploratory analyses, we conducted partial correlations to explore associations between alterations in hormone concentrations in acAN-T1 while controlling for BMI-SDS. This approach aimed to remove shared variance attributable to BMI-SDS between these parameters.

## 3. Results

### 3.1. Demographic, anthropometric and symptom variables of the study groups

As expected, the age distribution did not differ between the acAN and HC groups, and acAN-T1 exhibited significantly lower BMI-SDS and leptin concentrations, alongside higher levels of psychopathology (as indicated by EDI-2, BDI-II, and SCL-90-R scores) compared to HC (Table 1). While these parameters improved in the acAN-T2 group following short-term weight restoration (Table 1), residual psychopathology persisted if compared to HC.

### 3.2. Differences in parameters of glucose homeostasis and gut-secreted hormones in acAN (cross-sectional comparison)

**Fig. 1.**
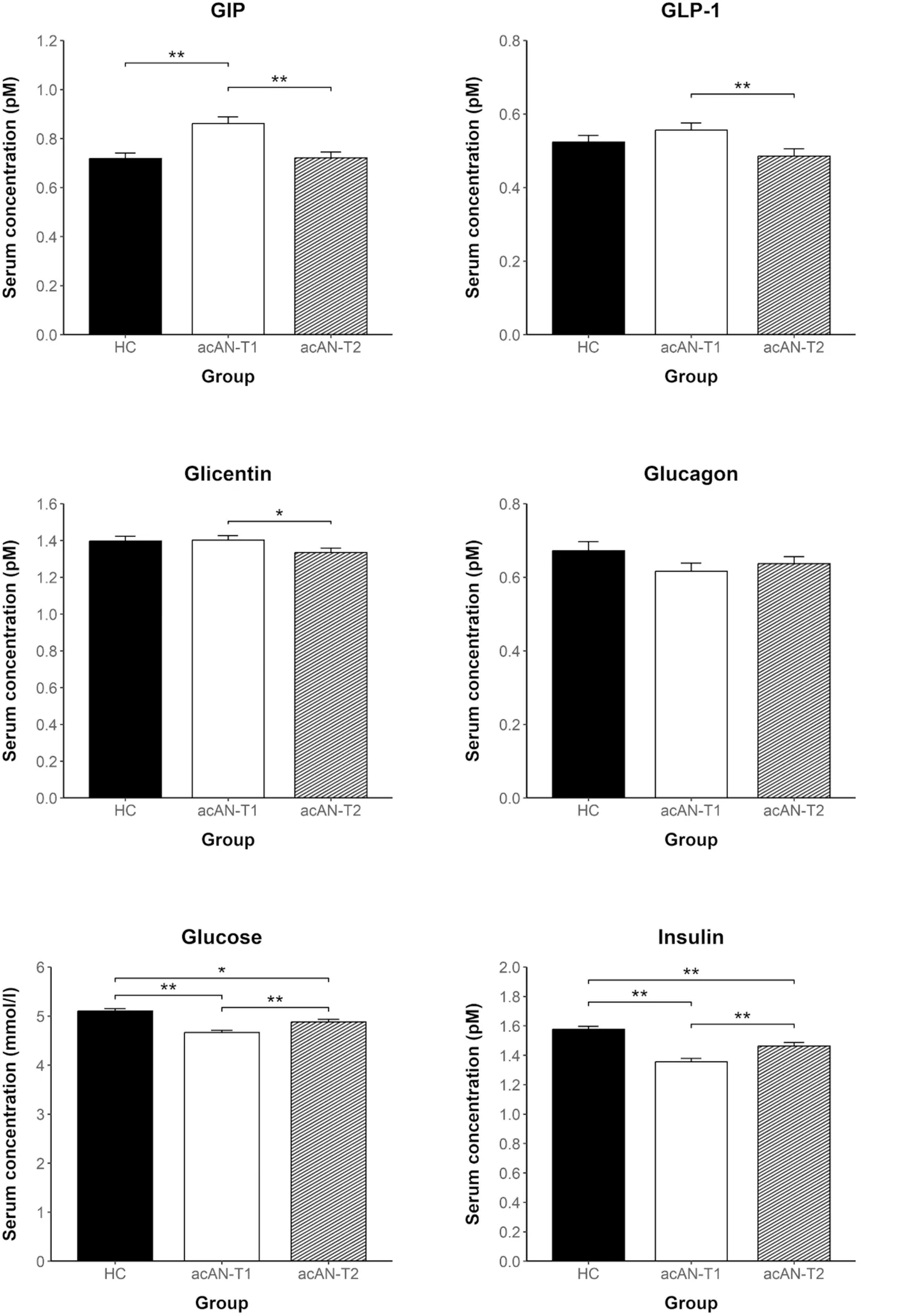
Group Comparison of Blood Parameters. Mean ± SEM for GIP, GLP-1, glicentin, glucagon, glucose, and insulin concentrations for HC, acAN-T1 and acAN-T2. All parameters, except glucose, were log10 transformed. Group comparisons were conducted using Student’s t-test: independent *t*-tests for HC vs. acAN-T1/acAN-T2, and paired t-tests for acAN-T1 vs acAN-T2. Asterisks indicate statistically significant differences *P < 0.05, **P < 0.01 after FDR correction. Abbreviations – acAN-T1, patients with anorexia nervosa in the acute state; acAN-T2, patients with anorexia nervosa after weight restoration; FDR, false discovery rate; GIP, gastric inhibitory peptide; GLP-1, glucagon-like peptide-1; HC, healthy controls.

Regarding our blood parameters of interest (see Fig. 1 and Supplemental Table 1), GIP levels were significantly increased (42.9%) in acAN-T1 in comparison to HC. We found no significant alteration in GLP-1, glicentin, and glucagon serum levels. Furthermore, acAN-T1 demonstrated significantly lower fasting serum glucose (−8.4%) and fasting serum insulin levels (−42.6%) compared to HC. HOMA-IR was significantly lower in acAN-T1 compared to HC.

### 3.3. Differences in parameters of glucose homeostasis and gut hormones after weight-restoration in acAN (longitudinal-analysis)

Longitudinally (see Fig. 1 and Supplemental Table 1), GIP (−30.4%), GLP-1 (−9.7%), and glicentin (−15,7%) levels decreased after short-term weight restoration, and only GIP normalized in acAN-T2 (when compared to HC), verified by Bayesian statistics (BF_01_ = 5.827). Glucagon concentration did not change significantly between time-points. Regarding our basic metabolic parameters, glucose (4.5%) and insulin levels (41.4%) increased significantly from acAN-T1 to acAN-T2. However, both glucose and insulin levels at acAN-T2 remained lower compared to the HC group (insulin −18.9%, and glucose −4.3%). Moreover, HOMA-IR levels in acAN-T2 were significantly increased compared to acAN-T1 and HOMA-IR was lower in acAN-T2 when compared to HC. All group differences were confirmed in supplementary non-parametric tests.

### 3.4. Correlations of the concentrations of gut hormones and their Delta changes after weight restoration with psychopathologic and metabolic parameters

Positive correlations were revealed for GIP with GLP-1, glicentin, BDI-II, EDI-2, and SCL-90-R, while its relationships with glucose, leptin, and BMI-SDS were negative in acAN-T1 (Table 2 and Supplemental Table 2). Controlling for BMI-SDS, GIP only kept its significant relationships with GLP-1, glicentin, BDI-II, and EDI-2 (Table 2 and Supplemental Table 3).

We computed change scores (Δ) to explore the relationships of changes in GIP, GLP-1, and glicentin from acAN-T1 to acAN-T2 among themselves and with basic metabolic blood parameters (glucose, insulin, leptin) and clinical measures (BMI-SDS, EDI-2, BDI-II, and SCL-90-R). Correlations are reported in Table 2 and Supplemental Table 4. For Δ-GIP, Spearman’s correlation revealed a positive relationship with Δ-GLP-1 and a negative relationship with Δ-glucose. Further, a positive relationship was found between Δ-GLP-1 and Δ-glicentin.

## 4. Discussion

The elevation of fasting GIP levels in acute AN and their normalization after weight restoration revealed in the current study implicate GIP in the underlying pathophysiological processes of AN. Together with GIP, we assessed serum concentrations of GLP-1, glucagon, and glicentin in AN patients both before and after short-term weight restoration, representing the first study to examine these hormones collectively, their interrelationships, and their associations with glucose homeostasis and psychopathology.

As stated above, the most important finding of our study is the higher fasting GIP concentration in acute AN. In healthy individuals, GIP levels increase three- to six-fold after nutrient intake, stimulating insulin secretion and thus contributing to the normalization of glucose levels (Drucker and Holst, 2023; Perakakis et al., 2019; Wewer Albrechtsen et al., 2023). However, during fasting, GIP exerts the opposite function by stimulating glucagon secretion, which increases glycogenolysis and gluconeogenesis to maintain normal blood glucose levels (Drucker and Holst, 2023; El and Campbell, 2020; Wewer Albrechtsen et al., 2023). Insulin normally inhibits glucagon secretion and the reduction of insulin levels during fasting in healthy individuals might also contribute to glucagon release (Andersen and Holst, 2022; Drucker and Holst, 2023; Wewer Albrechtsen et al., 2023). Patients with AN have depleted energy stores and are consequently susceptible to hypoglycaemia during fasting. In our study, fasting glucose concentrations were lower in AN compared to healthy controls, albeit still within the normal range. Interestingly, glucagon levels were not elevated, despite the higher GIP and lower insulin levels. This indicates that other known factors controlling glucagon secretion - such as various proteins (e.g., cholecystokinin, somatostatin, neurotensin, arginine-vasopressin, ghrelin), amino acids, fatty acids or neural signals (Wewer Albrechtsen et al., 2023) - might trigger changes in AN that counteract the stimulatory effects of high GIP/low insulin levels on glucagon secretion. Insufficient glucagon release in patients with AN might have important consequences on their ability to adapt to fasting. Specifically, it might affect hepatic glucose production, ketone body formation and thermogenesis, thus leading to more severe protein breakdown and muscle loss as well as to higher risk for hypoglycemia, exarcerbation of cold intolerance and fatigue, especially during fasting (Perakakis et al., 2025; Wewer Albrechtsen et al., 2023). This metabolic dysregulation may need to be considered when developing refeeding protocols for these patients and may necessitate closer metabolic monitoring during treatment. The insufficient glucagon release in combination with the lower insulin levels also explain the low HOMA-IR in AN observed in our study, which suggests high insulin sensitivity. Increased expression of proteins involved in insulin signalling may further enhance peripheral insulin action, as previously reported (Zambuzzi et al., 2025). On the other hand, during refeeding, metabolism shifts from lipid or protein utilization to carbohydrate use as the main energy source (Perakakis et al., 2025), leading to hepatic glycogen repletion, higher glucose levels and consequently increased insulin secretion. This metabolic shift is reflected in our study by the increase of HOMA-IR, although values remained within physiologic range.

Our study also revealed that endogenous GIP concentrations were inversely associated with BMI-SDS in the patient group. Additionally, we have previously shown that fasting GIP concentrations in an obese population were inversely associated with the activation of attention-related areas in the brain, such as the visual cortices of the occipital lobe and the parietal lobe, when viewing images of highly desirable/high caloric versus less desirable/low caloric food (Perakakis et al., 2021). This raises the question of whether the elevated GIP levels might aggravate AN and promote weight loss in patients with AN through effects on brain functions. This question is very difficult to answer since the role of GIP in regulating energy homeostasis via central mechanisms remains controversial (Liskiewicz and Müller, 2024). Both drug-induced GIP receptor (GIPR) agonism, as well as GIPR antagonism can profoundly reduce food intake and body weight in humans (Véniant et al., 2024). While the exact mechanisms of function are poorly understood, regarding GIPR agonism, it has been suggested that binding to GIPR in area postrema activates signalling that reaches hypothalamic nuclei and amygdala through glutamatergic projections, leading to decreased appetite (Liskiewicz and Müller, 2024). On the other hand, continuous agonism may lead to desensitization or cellular internalization of GIPR, resulting in functional antagonism. This could explain the (otherwise counter-intuitive) strong weight-loss effects of GIPR antagonists (Campbell, 2021). In any case, the elevated GIP levels in AN warrant further investigation, as they might be involved in appetite and/or glucose homeostasis in these patients. In the future, GIPR could be a potential target for the development of pharmacological interventions for AN.

The second key finding of our study is that weight restoration treatment led to normalization of GIP levels and to an increase in glucose and insulin concentrations. The negative correlation between changes in GIP concentrations and changes in glucose levels during weight restoration provide additional evidence for the involvement of GIP in metabolic regulation in AN. In contrast to GIP, fasting GLP-1 and glicentin levels were not significantly altered in AN compared to healthy controls. However, the levels of both hormones significantly decreased during weight restoration treatment. This result is somewhat unexpected, as our previous studies in 125 participants of different glycemic states (30 with diabetes, 65 with prediabetes, 30 with normal glucose tolerance) and 42 participants with morbid obesity undergoing bariatric surgery (9 gastric banding, 16 Roux-en-Y bypass, 17 vertical sleeve gastrectomy) did not show changes in fasting GLP-1 and glicentin concentrations when metabolic alterations occurred (Hoffmann et al., 2023; Perakakis et al., 2019). Specifically, progress of prediabetes to diabetes was characterized by a decrease only of the postprandial but not fasting levels of GLP-1 and glicentin concentrations (Hoffmann et al., 2023). Similarly, after bariatric surgery in patients with morbid obesity, only postprandial and not fasting GLP-1 and glicentin levels increased and were positively associated with weight loss in these patients (Perakakis et al., 2019). The decreasing fasting GLP-1 and glicentin levels during weight restoration treatment in the current study did not correlate with glucose, insulin, or BMI improvement in these patients. Nevertheless, it is improbable that this finding is an artefact, since the concentrations of GLP-1 and glicentin with each other were very strongly correlated. This is consistent with our previous studies showing strong associations between these two hormones due to their co-secretion from the L-cells of the gut (Hoffmann et al., 2023; Perakakis et al., 2019; Perakakis et al., 2022b). Thus, we speculate that the reduction of GLP-1 and glicentin might result from the sudden increase in the exposure of L-cells to nutrients during weight restoration treatment. This sudden exposure might affect the secretion profile by prioritizing postprandial responses over “basal” secretion during fasting. Supporting this hypothesis, a previous study reported stronger postprandial increases in GLP-1 and glucagon after a 2-week refeeding period in AN. In the same study, elevated fasting values of glucagon, GIP, and GLP-1 tended to decrease in AN, though these changes did not reach statistical significance, likely due to the much smaller sample size compared to our study (Heruc et al., 2018). Notably, apart from glucose metabolism and appetite regulation, both hormones are involved in gastrointestinal motility, which is impaired in AN (Norris et al., 2016). Patients with AN are known to have a delayed gastric emptying, which improves during refeeding (Heruc et al., 2018; Norris et al., 2016). The fact that GLP-1 and glicentin levels did not decrease in the acute stage but following weight restoration distinguishes them from GIP. This distinct temporal pattern may reflect a different aspect of metabolic recovery and suggests that these hormones could serve as biomarkers for long-term clinical outcome in AN. Future studies should assess whether the reduction in fasting GLP-1 and glicentin levels has any impact on the course of AN and weight restoration treatment.

Finally, a third interesting finding in our study was the positive association of GIP levels with symptom levels, such as depressive (BDI-II), ED (EDI-2), and overall (SCL-90-R) symptoms. This relationship was only partially dependent on BMI, suggesting GIP is associated with these symptoms beyond the effect of BMI. Although to date no other studies have directly linked GIP to mental health conditions or respective symptoms, similar future research could offer some valuable insights to this relationship. GIP levels are often elevated in obesity and the metabolic syndrome, both of which are associated with alterations in mood and an increased risk of depressive symptoms (Bădescu et al., 2016; Fulton et al., 2022; Toalson et al., 2004).This may be linked to the dysregulation of hormones such as GIP. Such, preliminary, evidence supports the idea that GIP could be a target for the treatment of AN.

Our study has to be considered in the light of its strengths and limitations. The strengths include the large sample size and the use of highly accurate, modern assays which reduced the likelihood of cross-reactivity. Our major limitation is the absence of postprandial concentration assessments, such as those following an oral glucose tolerance test (OGTT) or a mixed meal test. Nevertheless, considering that patients with AN typically remain in a fasting state for the vast majority of the day, evaluating hormone levels after overnight fasting is also highly relevant to understanding the metabolic state of this disorder. However, future studies could provide a more comprehensive view of the metabolic state in AN by adding postprandial measurements. Another limitation to consider is the demographic composition of our sample, which consists solely of female participants. Thus, a generalization of our results to male patients with AN is not possible.

In conclusion, our study highlights the role of elevated fasting GIP concentrations in AN and their potential involvement in the dysregulation of glucose homeostasis as well as associations with symptom severity. The normalization of GIP levels with weight restoration treatment and the associated changes in glucose and insulin concentrations provide additional support to the importance of GIP in metabolic regulation in individuals with AN. However, our unexpected findings regarding GLP-1 and glicentin levels during weight restoration treatment implicate rather complex alterations in gut hormones, likely due to the changes in nutrients and gastrointestinal motility. Future studies should explore these dynamic alterations further by incorporating postprandial measurements for a more comprehensive understanding of the metabolic mechanisms in AN. Based on the findings of the current study, further research could strengthen the case for targeting GIP in drug development for the treatment of AN, as it is linked to symptoms – particularly ED symptoms - above and beyond the effect of BMI. These findings highlight the complex nature of hormonal dysregulation in AN and suggest that GIP may have a more central role than previously recognized.

## Supplementary Material

1

## Figures and Tables

**Table 1:** Demographic and clinical characteristics.

	Missing	HC (*N* = 80)	acAN-T1 (N = 80)	acAN-T2 (*N* = 79)	Significant group differences	*p*-value

**Age (years)**	/	16.05 (2.67)	14.90 (2.05)	15.90 (2.10)	*acAN-T1 < acAN-T2*	< 0.001
**IQ**	*N* = 3	113.00 ± 9.67	110.90 ± 13.31	/	/	
**BMI (kg/m^2^)**	/	20.61 ± 2.30	14.90 ± 1.21	19.04 ± 1.12	*acAN-T1 < HC, acAN-T2 < HC, acAN-T1 < acAN-T2*	< 0.001
						< 0.001
						< 0.001
**BMI-SDS**	/	−0.04 ± 0.75	−2.94 ± 0.99	−0.60 ± 0.49	*acAN-T1 < HC, acAN-T2 < HC, acAN-T1 < acAN-T2*	< 0.001
						< 0.001
						< 0.001
**Minimal Lifetime BMI (kg/m^2^)**	*N* = 11	19.89 ± 1.94	14.63 ± 1.26	/	*acAN-T1 < HC*	< 0.001
**HOMA-IR**	/	1.20 (0.73)	0.64 (0.43)	0.93 (0.54)	*acAN-T1 < HC, acAN-T2 < HC, acAN-T1 < acAN-T2*	< 0.001
						< 0.001
						< 0.001
**BDI-II (total score)**	*N* = 10	3.00 (5.99)	23.00 (15.00)	13.00 (14.00)	*acAN-T1 > HC, acAN-T2 > HC, acAN-T1 > acAN-T2*	< 0.001
						< 0.001
						< 0.001
**EDI-2 (total score)**	*N* = 12	139.36 ± 28.11	210.50 ± 44.09	197.49 ± 49.37	*acAN-T1 > HC, acAN-T2 < HC, acAN-T1 > acAN-T2*	< 0.001
						< 0.001
						0.001
**SCL-90-R (total score)**	N = 8	18.00 (27.25)	78.00 (67.00)	50.50 (58.75)	*acAN-T1 > HC, acAN-T2 > HC, acAN-T1 < acAN-T2*	< 0.001
						< 0.001
						< 0.001
**Leptin (ng/ml)**	*N* = 9	7.78 (9.55)	0.95 (2.06)	11.60 (9.75)	*acAN-T1 > HC, acAN-T2 > HC, acAN-T1 < acAN-T2*	< 0.001
						0.014
						< 0.001

*Note.* HC = healthy controls; acAN-T1 = patients with anorexia nervosa in the acute state at timepoint 1; acAN-T2 = patients with anorexia nervosa after partial weight restoration at timepoint 2. Units: kg/m^2^ = kilogram/square meter; ng/ml = nanogram/ml. Mean ± standard deviation reported for parameters with normal distribution. Median (interquartile range) reported for non-normally distributed data. Missing values reported across all groups. Parametric group comparisons have been examined through Student’s *t*-test (paired for acAN-T1 with acAN-T2; unpaired for HC with acAN-T1, and HC with acAN-T2), non-parametric group comparisons have been examined through Mann-Whitney-*U* test (for HC with acAN-T1, and HC with acAN-T2) or Wilcoxon Signed-Ranked Text (for acAN-T1 with acAN-T2).

**Table 2:** Correlations between hormonal changes and clinical parameters in patients with AN before and after weight-restoration.

(A)											
										
	GLP-1	Glicentin	Glucagon	Glucose	Insulin	Leptin	BMI-SDS	BDI-II	EDI-2	SCL-90-R	

	*alterations in acAN-T1*										
**GIP**	0.254**	0.227**	−0.008	−0.221*	−0.152	−0.249**	−0.225*	0.278*	0.290**	266**	
	*alterations in acAN-T1 adjusted for BMI-SDS*										
**GIP**	0.272**	0.233**	−0.014	−0.125	−0.056	−0.023	–	0.195*	0.220*	0.144	
(B)											
	GIP	GLP-1	Glicentin	Glucagon	Glucose	Insulin	Leptin	BMI-SDS	BDI-II	EDI-2	SCL-90-R
	*change scores between acAN-T1 and acAN-T2*										
**GIP**	–	0.301*	0.210	0.084	−0.279*	−0.136	0.034	−0.053	−0.022	0.068	0.069
**GLP-1**		–	0.812**	0.135	−0.179	−0.055	−0.025	−0.191	−0.017	−0.147	−0.080
**Glicentin**			–	0.151	−0.203	−0.070	0.069	−0.114	−0.110	−0.253	−0.132

*Note.* (A) Spearman correlations of values of healthy controls (HC) and anorexia nervosa patients in the acute state (acAN-T1) with adjustment for BMI-SDS through partial correlation. (B) Spearman correlation matrix of change scores in parameters between acAN-T1 and after short-term restoration (acAN-T2). Asterisks ** and * denote statistical significance respectively at the 1% and 5% level. *P*-values were adjusted for multiple comparisons using False Discovery Rate (FDR) correction method (Benjamini and Hochberg, 1995). GIP (pM), GLP-1 (pM), glicentin (pM), and insulin (pM) values were log10 transformed before correlation. Glucose in mmol/l, leptin in ng/ml. Total scores for BDI-II and EDI-2, and global severity index for SCL-90-R.

## Data Availability

Data will be made available on request.

## Appendix A. Supplementary data

Supplementary data to this article can be found online at https://doi.org/10.1016/j.pnpbp.2025.111576.

## Abbreviations:

acAN: patients with acute anorexia nervosa
acAN-T1: patients with anorexia nervosa at the acutely underweight state
acAN-T2: patients with anorexia nervosa after short-term weight restoration
AN,: anorexia nervosa
BDI-II: Beck-Depression-Inventar Revision
BMI-SDS: body mass index standard deviation score
ED: eating disorder
EDI-2: Eating Disorder Inventory-2
FDR: false discovery rate
GIP: gastric inhibitory peptide
GIPR: drug-induced gastric inhibitory peptide receptor
GLP-1: glucagon-like peptide-1
HC: healthy controls
IQ: intelligence quotient
OGTT: oral glucose tolerance test
SCL-90-R: Symptom-Checklist
SIAB-EX: Structured Interview for Anorexia and Bulimia Nervosa
T2DM: type 2 diabetes mellitus
