# Supplementary material for: Circulating levels of gut hormones in anorexia nervosa before and after short-term weight restoration
Source: Prog Neuropsychopharmacol Biol Psychiatry. Author manuscript; Available in PMC 2026 Aug 31. (PMC13527557; doi:10.1016/j.pnpbp.2025.111576)
Supplement: Supplementary file 1 [file NIHMS2189445-supplement-1.docx]

# Supplementary Material

## 1.1. Correlations of the concentrations of gut hormones and their Delta changes after weight recovery with psychopathologic and metabolic parameters

Controlling for BMI-SDS, for insulin only the correlation with leptin remained significant.

To explore the relationships of changes from acAN-T2 to acAN-T2 in glucose and insulin with leptin, BMI-SDS, EDI-2, BDI-II, and SCL-90-R, change scores (Δ) were computed for these parameters. When controlling for Δ-BMI-SDS, a negative relationship between Δ-insulin and Δ-EDI-2 was revealed.

**Supplemental Table 1.** Concentrations of blood parameters of glucose homeostasis and gut-secreted hormones.

|  | **HC**^1,3^  **(N=80)** | **acAN-T1**^1,3^  **(N=80)** | **acAN-T2**^1,3^  **(N=79)** | **Significant group**  **comparisons**^3^ | **Unadjusted**  **p-value** | **FDR**^1,3^  **q-value** |
| --- | --- | --- | --- | --- | --- | --- |
| **Glucose (mmol/l)** | 5.1 ± 0.45 | 4.67 ± 0.4 | 4.88 ± 0.49 | *HC > acAN-T1,*  *acAN-T1 > acAN-T2,*  *HC > acAN-T2* | <.001  <.001  .003 | **<.001**  **.003**  **.013** |
| **Insulin (pM)** | 37.19 (21.07) | 21.33 (14.77) | 30.15 (17.05) | *HC > acAN-T1,*  *acAN-T1 > acAN-T2,*  *HC > acAN-T2* | <.001  <.001  .001 | **<.001**  **.002**  **.003** |
| **GIP (pM)** ^1,2^ | 5.04 (3.73) | 7.2 (4.61) | 5.01 (2.27) | *acAN-T1 > HC,*  *acAN-T1 > acAN-T2* | <.001  <.001 | **.001**  **<.001** |
| **GLP-1 (pM)** ^1,2^ | 3.5 (1.64) | 3.6 (1.15) | 3.25 (1.59) | *acAN-T1 > acAN-T2* | .001 | **.005** |
| **Glicentin (pM)** | 25.23 (18.9) | 25.56 (17.14) | 21.54 (15.42) | *acAN-T1 > acAN-T2* | .014 | **.039** |
| **Glucagon (pM)** | 4.28 (3.0) | 4.2 (1.94) | 4.34 (2.1) | */* | / | / |

^1^acAN-T1, patients with anorexia nervosa in the acute state; acAN-T2, patients with anorexia nervosa after weight restoration; FDR, false discovery rate; GIP, gastric inhibitory peptide; GLP-1 glucagon-like peptide-1; HC, healthy controls.

^2^Units: pM=picomolar; mmol/L=millimoles/liter.

^3^Mean ± standard deviation reported for parameters with normal distribution. Median (interquartile range) reported for non-normally distributed data. Missing values in glucose at T1 (N=1) and T2 (N=2). Group comparisons have been examined through Student’s t-test (paired for acAN-T1 with acAN-T2; unpaired for HC with acAN-T1, and HC with acAN-T2) on glucose and log10 transformed data for insulin, GIP, GLP-1, glicentin, glucagon. P-values were adjusted for multiple comparisons using FDR correction method (Benjamini & Hochberg, 1995), values printed in bold indicate significance after FDR correction.

**Supplemental Table 2.** Spearman’s rank correlation coefficient (Spearman’s rho) for alterations in blood parameter concentrations and clinical parameters in the acAN-T1 group. ^1^

|  | **Glucose**^2,3^ | **Insulin**^2,3^ | **GIP**^1,2,3^ | **GLP-1**^1, 2,3^ | **Glicentin**^2,3^ | **Glucagon**^2,3^ | **Leptin**^2,3^ | **BMI-**  **SDS**^1,3^ | **BDI-II**^1, 2,3^ | **EDI-2**^1,2,3^ |
| --- | --- | --- | --- | --- | --- | --- | --- | --- | --- | --- |
| **Glucose** | − |  |  |  |  |  |  |  |  |  |
| **Insulin** | .441** | − |  |  |  |  |  |  |  |  |
| **GIP** | -.221* | -.152 | − |  |  |  |  |  |  |  |
| **GLP-1** | -.115 | -.099 | .254** |  |  |  |  |  |  |  |
| **Glicentin** | .046 | -.117 | .227** | .462** |  |  |  |  |  |  |
| **Glucagon** | -.139 | .080 | -.008 | .286** | .107 |  |  |  |  |  |
| **Leptin** | .519** | .580** | -.249** | -.151 | -.070 | .107 | − |  |  |  |
| **BMI-SDS** | .500** | .527** | -.225** | -.109 | -.040 | .060 | .835** | − |  |  |
| **BDI-II** | -.356** | -.293** | .278** | .036 | -.056 | -.081 | -.506** | -.617** | − |  |
| **EDI-2** | -.301** | -.270** | .290** | -.023 | -.010 | -.098 | -.425** | -.525** | .878** | − |
| **SCL-90-R**^1,2,3^ | -.314** | -.270** | -266** | .039 | -.059 | -.123 | -.443** | -.535** | .885** | .842** |

^1^ acAN-T1, patients with anorexia nervosa in the acute state; BDI-II, Beck-Depression-Inventar Revision; BMI-SDS, body mass index standard deviation score; EDI-2, Eating Disorder Inventory-2; FDR, false discovery rate; GIP, gastric inhibitory peptide; GLP-1 glucagon-like peptide-1; SCL-90-R, Symptom-Checklist.

^2^ GIP (in pM, picomolar), GLP-1 (pM), glicentin (pM), and insulin (pM) values were log10 transformed before correlation. Glucose in mmol/L, millimoles per liter; leptin in ng/ml, nanograms per milliltre. Total scores for BDI-II and EDI-2, and global severity index for SCL-90-R.

^3^ Correlation coefficients are reported below the diagonal. Asterisks ** and * denote statistical significance (2-tailed) respectively at the 1% and 5% level. P-values were adjusted for multiple comparisons using FDR correction method (Benjamini & Hochberg, 1995).

**Supplemental Table 3.** Partial correlation coefficient for alterations in blood parameter concentrations and clinical parameters in the acAN-T1 adjusted for Δ-BMI-SDS. ^1^

|  | | **Glucose**^2,3^ | **Insulin**^2,3^ | **GIP**^1,2,3^ | **GLP-1**^1, 2,3^ | **Glicentin**^2,3^ | **Glucagon**^2,3^ | **Leptin**^2,3^ | **BDI-II**^1, 2,3^ | **EDI-2**^1,2,3^ |  |
| --- | --- | --- | --- | --- | --- | --- | --- | --- | --- | --- | --- |
| **Glucose** | | − |  |  |  |  |  |  |  |  |  |
| **Insulin** | | .251** | − |  |  |  |  |  |  |  |  |
| **GIP** | | -.125 | -.056 | − |  |  |  |  |  |  |  |
| **GLP-1** | | -.081 | -.130 | .272** |  |  |  |  |  |  |  |
| **Glicentin** | | .087 | -.167* | .233** | .363** |  |  |  |  |  |  |
| **Glucagon** | | -.186* | .001 | -.014 | .393** | .066 |  |  |  |  |  |
|  | **Leptin** | .190* | .249** | -.023 | .013 | .033 | .070 | − |  |  | |
| **BDI-II** | | -.017 | .056 | .195* | -.024 | -.068 | -.110 | .042 | − |  |  |
| **EDI-2** | | -.004 | .080 | .220** | -.049 | -.017 | -.111 | .115 | .843** | − |  |
| **SCL-90-R**^1,2,3^ | | .003 | .073 | .144 | .004 | -.038 | -.108 | .061 | .840** | .768** |  |

^1^ acAN-T1, patients with anorexia nervosa in the acute state; acAN-T2, patients with anorexia nervosa after weight restoration; BDI-II, Beck-Depression-Inventar Revision; BMI-SDS, body mass index standard deviation score; EDI-2, Eating Disorder Inventory-2; FDR, false discovery rate; GIP, gastric inhibitory peptide; GLP-1 glucagon-like peptide-1; HC, healthy controls; SCL-90-R, Symptom-Checklist.

^2^ GIP (in pM, picomolar), GLP-1 (pM), glicentin (pM), and insulin (pM) values were log10 transformed before correlation. Glucose in mmol/L, millimoles per liter; leptin in ng/ml, nanograms per milliltre. Total scores for BDI-II and EDI-2, and global severity index for SCL-90-R.

^3^ Correlation coefficients are reported below the diagonal. Asterisks ** and * denote statistical significance (2-tailed) respectively at the 1% and 5% level. P-values were adjusted for multiple comparisons using FDR correction method (Benjamini & Hochberg, 1995).

**Supplemental Table 4.** Spearman’s rank correlation coefficient (Spearman’s rho) for change scores between acAN-T1 and acAN-T2 with clinical parameters.

|  | Δ-**Glucose**^2,3^ | Δ-**Insulin**^2,3^ | Δ-**GIP**^1,2,3^ | Δ-**GLP-1**^1,2,3^ | Δ-**Glicentin**^2,3^ | Δ-**Glucagon**^2,3^ | Δ-**Leptin**^2,3^ | Δ-  **BMI-SDS**^1,3^ | Δ-  **BDI-II**^1,2,3^ | Δ-  **EDI-2**^1,2,3^ |
| --- | --- | --- | --- | --- | --- | --- | --- | --- | --- | --- |
| Δ-**Glucose** | − |  |  |  |  |  |  |  |  |  |
| Δ-**Insulin** | .334** | − |  |  |  |  |  |  |  |  |
| Δ-**GIP** | -.279* | -.136 | − |  |  |  |  |  |  |  |
| Δ-**GLP-1** | -.179 | -.055 | .301** | − |  |  |  |  |  |  |
| Δ-**Glicentin** | -.203 | -.070 | .210 | .812** | − |  |  |  |  |  |
| Δ-**Glucagon** | .083 | .170 | .084 | .135 | .151 |  |  |  |  |  |
| Δ-**Leptin** | .008 | .162 | .034 | -.025 | .069 | .179 | − |  |  |  |
| Δ-**BMI-SDS** | .193 | .135 | -.053 | -.191 | -.114 | .050 | .218 | − |  |  |
| Δ-**BDI-II** | -.105 | .023 | -.022 | -.017 | -.110 | -.209 | -.092 | .015 | − |  |
| Δ-**EDI-2** | -.145 | -.187 | .068 | -.147 | -.253** | -.272* | -.053 | .045 | .528** | − |
| Δ- **SCL-90-R**^1,2,3^ | -.170 | .008 | .069 | -.080 | -.132 | -.327** | -.091 | .057 | .633** | .515** |

^1^ acAN-T1, patients with anorexia nervosa in the acute state; BDI-II, Beck-Depression-Inventar Revision; BMI-SDS, body mass index standard deviation score; EDI-2, Eating Disorder Inventory-2; FDR, false discovery rate; GIP, gastric inhibitory peptide; GLP-1 glucagon-like peptide-1; SCL-90-R, Symptom-Checklist.

^2^ GIP (in pM, picomolar), GLP-1 (pM), glicentin (pM), and insulin (pM) values were log10 transformed before correlation. Glucose in mmol/L, millimoles per liter; leptin in ng/ml, nanograms per milliltre. Total scores for BDI-II and EDI-2, and global severity index for SCL-90-R.

^3^ Correlation coefficients are reported below the diagonal. Asterisks ** and * denote statistical significance (2-tailed) respectively at the 1% and 5% level. P-values were adjusted for multiple comparisons using FDR correction method (Benjamini & Hochberg, 1995).
